# Sphingolipid Catabolism and Glycerophospholipid Levels Are Altered in Erythrocytes and Plasma from Multiple Sclerosis Patients

**DOI:** 10.3390/ijms23147592

**Published:** 2022-07-08

**Authors:** Albena Momchilova, Roumen Pankov, Alexander Alexandrov, Tania Markovska, Stefan Pankov, Plamen Krastev, Galya Staneva, Evgenia Vassileva, Nikolai Krastev, Adriana Pinkas

**Affiliations:** 1 Institute of Biophysics and Biomedical Engineering, Bulgarian Academy of Sciences, Acad. G. Bonchev Str. Bl. 21, 1113 Sofia, Bulgaria; asalexandrov@abv.bg (A.A.); markobska@abv.bg (T.M.); stefan.pankov@abv.bg (S.P.); g_staneva@yahoo.com (G.S.); 2 Biological Faculty, Sofia University, 8, Dragan Tzankov Str., 1164 Sofia, Bulgaria; rpankov@biofac.uni-sofia.bg; 3 Cardiology Clinic, University Hospital St. Ekaterina, 1431 Sofia, Bulgaria; plamenkr@mail.bg; 4 Clinic of Neurology, Tsaritsa Yoanna University Hospital-ISUL, 1527 Sofia, Bulgaria; e.vassilevva@gmail.com; 5 Department of Anatomy, Histology and Embryology, Medical University-Sofia, Blvd. Sv. Georgi Sofiisky 1, 1431 Sofia, Bulgaria; dr.krustev.dm@gmail.com; 6 Medical Center Relax, 8 Ami Bue Str., 1606 Sofia, Bulgaria; 7 STEP/CSTEP, Office of Continuing Education, Suffolk County Community College 30 Greene Ave., Sayville, NY 11782, USA; asarafova@yahoo.com

**Keywords:** multiple sclerosis, sphingolipids, ceramide, sphingosine, acid sphingomyelinase, alkaline ceramidase, fatty acids, oxidative stress

## Abstract

Multiple sclerosis (MS) is an autoimmune, inflammatory, degenerative disease of the central nervous system. Changes in lipid metabolism have been suggested to play important roles in MS pathophysiology and progression. In this work we analyzed the lipid composition and sphingolipid-catabolizing enzymes in erythrocytes and plasma from MS patients and healthy controls. We observed reduction of sphingomyelin (SM) and elevation of its products—ceramide (CER) and shingosine (SPH). These changes were supported by the detected up-regulation of the activity of acid sphingomyelinase (ASM) in MS plasma and alkaline ceramidase (ALCER) in erythrocytes from MS patients. In addition, Western blot analysis showed elevated expression of ASM, but not of ALCER. We also compared the ratios between saturated (SAT), unsaturated (UNSAT) and polyunsaturated fatty acids and suggest, based on the significant differences observed for this ratio, that the UNSAT/SAT values could serve as a marker distinguishing erythrocytes and plasma of MS from controls. In conclusion, the application of lipid analysis in the medical practice would contribute to definition of more precise diagnosis, analysis of disease progression, and evaluation of therapeutic strategies. Based on the molecular changes of blood lipids in neurodegenerative pathologies, including MS, clinical lipidomic analytical approaches could become a promising contemporary tool for personalized medicine.

## 1. Introduction

Multiple sclerosis (MS) is an immune-mediated, neurodegenerative, demyelinating, chronic disease of unknown etiology with a possible genetic predisposition and effect of certain environmental factors [1,2]. This neurological disease with a wide variety of symptoms and clinical manifestations often leads to serious damage of the motor activity, paresis and paralysis, disturbed vision, disorders in the function of the pelvic organs, etc. [3,4]. Recent reports highlighted the potential usefulness of lipid markers in prediction or monitoring the course of MS particularly in its progressive stages, which is still insufficiently addressed [5]. Changes in lipid metabolism and separate lipid molecular species have been suggested to play important roles in multiple sclerosis pathophysiology and pathogenesis, and affect disease progression. Sphingolipids, together with glycerophospholipids are involved in many cellular functions, including cell proliferation, signaling cascades, apoptosis, participation in pro- or anti-proliferative pathways, etc. [6]. Especially sphingolipids, which are largely expressed in the central nervous system (CNS) and display multiple biological functions, have been closely implicated in the pathogenesis of MS [7]. The most functionally active members of the sphingolipid (SL) family are sphingomyelin (SM), ceramide (CER), sphingosine (SPH) and sphingosine-1-phosphate (S1P) [7]. S1P participates in the pathogenesis of various pathological processes and diseases, including inflammation, oxidative stress, neurodegenerative pathologies such as MS, etc. [8]. CER is involved in the progression of various neurodegenerative pathologies [9].

CERs with short carbon chains were found to stimulate oxygen species accumulation in hippocampal glial cells, which leads to oxidative stress and neuronal death [7,10,11]. TNF-α mediated signaling is related to activation of phospholipase A2 (PLA2) and release of arachidonic acid (AA), the latter inducing activation of sphingomyelinases that hydrolyze SM to produce CER [8]. This mechanism underlies the imbalance of cytokines which in turn affects phospholipid (PL) and sphingolipid (SL) metabolism [12].

The other member of the SL family, SPH, has received less research attention, compared to CER and S1P. However, the role of SPH in degeneration of oligodendrocytes and neurons in MS patients has been documented [13]. There are also reports illustrating the devastating role of SPH in CNS demyelination in MS progression [14]. To illustrate the specific pathogenic role of sphingolipids in MS, Dasgupta and Ray reported elevated concentrations of CER and SPH in CNS, this leading to demyelination and oligodendrocyte death [15]. In addition, inflammation processes stimulate SPH accumulation, thus contributing to demyelination and neurodegeneration [16].

Oxidative stress is harmful in MS and results in formation of oxidized lipid products. Lipid peroxidation plays significant role in MS pathogenesis which is associated with stimulation of apoptotic events [17]. Polyunsaturated fatty acids (PUFA) are an excellent target of oxidative attack and the oxidized products interact and destroy cell membranes and myelin sheaths. Lipid peroxidation products, such as malondialdehyde (MDA) and isoprostanes, react with other molecules and thus contribute to oxidative stress expansion and inflammation in the CNS [18].

Reports show augmentation of lipid peroxidation products in cerebrospinal fluid (CSF) and plasma of MS patients [18]. Accumulation of high levels of isoprostanes, MDA and other oxidative stress markers have been reported in plasma, serum, brain and CSF [19]. Lipid peroxidation correlates with intensive demyelination and neuro-degeneration in multiple sclerosis. Oxidized products of phosphatidylcholine are elevated in MS plaques and have been suggested to serve as a marker for neuro-inflammation in MS brain tissue [20]. Much attention has been focused on the role of oxidative stress in the pathogenesis of MS [21,22,23]. Excessive generation of ROS has been reported to induce myelin loss and nerve tissue degeneration [24]. The high levels of ROS react readily with cellular and extracellular molecules, including proteins and lipids, thus provoking oxidative damage to these molecules and changing their functional activity.

Erythrocytes usually have bigger diameters in MS, which is positively correlated with the progression of the disease. The erythrocytes lipid content, lipid metabolism and molecular profile are different in MS patients, which makes these cells an object of special interest. Red blood cells are assumed as a major store and source of S1P, which, as already mentioned, plays a specific pathogenic role in MS. Since S1P has been largely studied due to its ubiquitous functional activity, in this work we focused our attention on the mechanism of accumulation of two other important sphingolipid metabolites—CER and SPH, the latter being recognized as a precursor of S1P.

Studies were carried out on the SM–S1P pathway and were focused particularly on CER and SPH and on the enzyme activities responsible for their accumulation—sphingomyelinase and ceramidase. As already mentioned, the SM signaling cascade is composed mainly of SM, CER, SPH and S1P, in which one component emerges from the other by sequential enzymatic degradation. The obtained results showed that erythrocytes and plasma of MS patients contain less SM and PUFA, as well as higher levels of CER and SPH, compared to controls. Also, blood plasma of MS patients was observed to contain upregulated sphingomyelinase (SMase) activity, which we presume is responsible for the reduction of SM, the latter acting as a natural membrane antioxidant [25].

## 2. Results

### 2.1. Patients Characteristics

The data showing the clinical course of MS according to Kurtzke’s Expanded Disability Status Scale (EDSS) for the eighteen patients under investigation are shown in Table 1. The EDSS values varied in the range 4–7. Patients 1–9 were male and patients 10–18 were female. The patients involved in this study were from Clinic of Neurology, Tsaritsa Yoanna University Hospital-ISUL and Medical Center Relax. Blood was drawn from all patients during remission phase. We selected patients with compatible parameters such as no disease-modifying therapy, no diet restrictions, non-smokers and no other registered diseases except MS.

The characteristics of the patients and the controls are presented in Table 2. Besides the 9 male and 9 female patients, the study involved as well as 9 male and 9 female control individuals.

### 2.2. Phospholipid Analysis of Erythrocyte Membranes and Plasma from Multiple Sclerosis Patients

**Figure 1:**
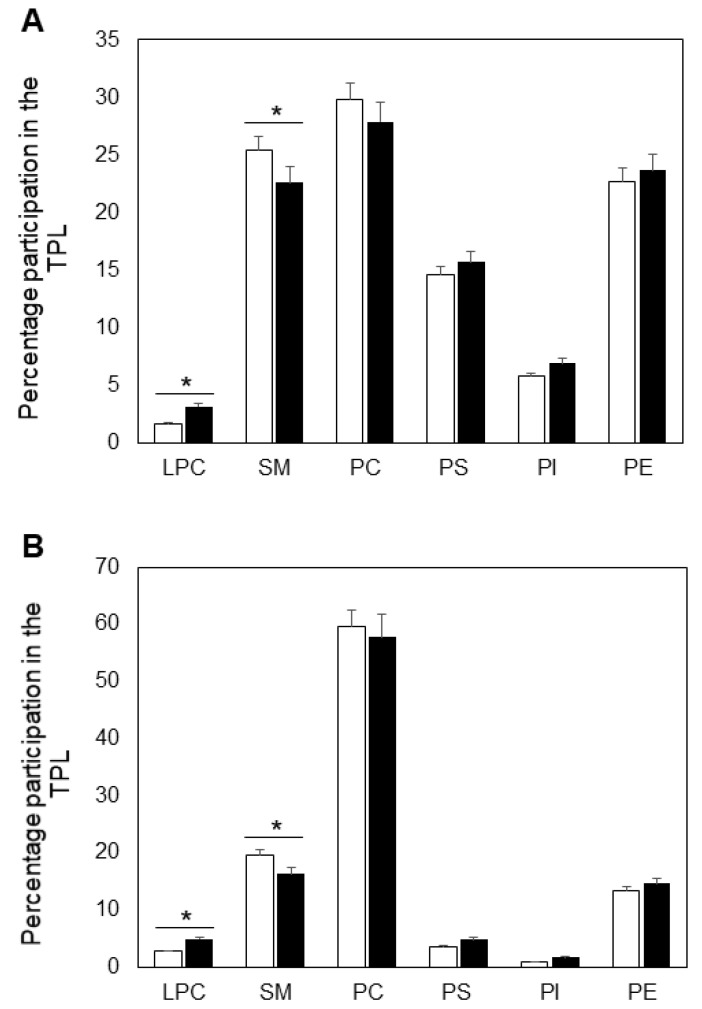
Phospholipid composition of erythrocyte ghosts (**A**) and plasma (**B**) from multiple sclerosis patients (filled bars) and control individuals (empty bars). Values are expressed as relative percentage participation in the total phospholipids (TPL). All measurements were performed in triplicate. Values are means ± SD. * *p* < 0.05.

In this work we studied the alterations in the phospholipid (PL) composition of erythrocyte membranes (ghosts) and plasma form patients with MS (Figure 1). We were interested especially in the changes of the level and metabolism of SM and its metabolites, because these lipids are implicated in the pathophysiology and progression of MS [7]. Figure 1A shows the changes in the PL composition (presented as percentage participation in the total PL) of erythrocyte membranes isolated from blood samples of MS patients and control individuals. SM was decreased by 12% in erythrocyte membranes of MS patients, compared to controls. In addition, the other choline-containing PL, phosphatidylcholine (PC), was also reduced in MS erythrocytes but this reduction was not statistically significant. It should be noted that the amino PLs phosphatidylethanolamine (PE) and phosphatidylserine (PS) were slightly elevated MS ghosts, but again the observed differences were statistically insignificant.

Since there is permanent lipid exchange between erythrocytes and plasma, we also analyzed the PL composition of plasma from MS and healthy individuals (Figure 1B). In MS plasma we also observed a decrease of the choline-containing PLs, PC and SM, the reduction of the latter being statistically significant Again, there was an insignificant increase in the level of the two amino-PLs, PE and PS.

It should be noted that in plasma and ghosts of both analyzed groups we tested the phospholipid, lysophosphatidylcholine (LPC) that is missing in most of the reports devoted to blood PL composition in MS patients. Since LPC, which was elevated in MS patients, is a hydrolytic product of PC, we were interested in analyzing of the phospholipase activity responsible for degradation of PC to LPC and arachidonic acid (AA) which is discussed below.

### 2.3. Acid Sphingomyelinase (ASM), Alkaline Ceramidase (ALCER) and Phospholipase A2 (PLA2) Activity

**Figure 2:**
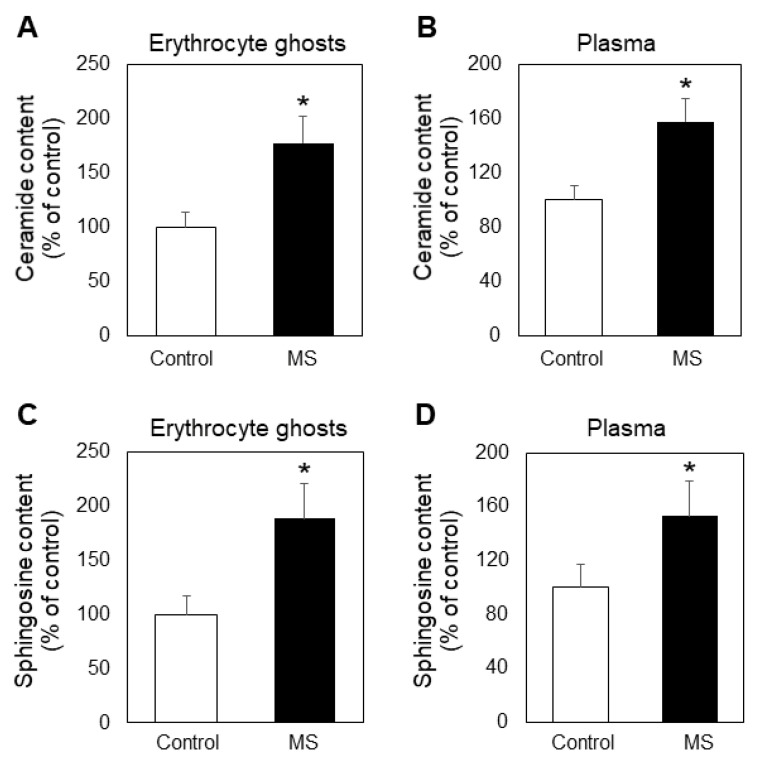
Alterations in the level of ceramide in erythrocyte ghosts (**A**) and plasma (**B**) and sphingosine in erythrocyte ghosts (**C**) and plasma (**D**) from multiple sclerosis (MS) patients (filled bars) and control individuals (empty bars). Values are expressed as % of control (100%). All measurements were performed in triplicate. Values are means ± SD. * *p* < 0.05.

The reduction of SM is an interesting finding, which deserves special attention. SM is the source of the functionally active CER and SPH, which have been related to demyelination and oligodendrocyte death in MS [15]. We found that concomitant with SM decrease, both of these intermediate sphingolipids were elevated in MS ghosts and plasma (Figure 2).

**Figure 3:**
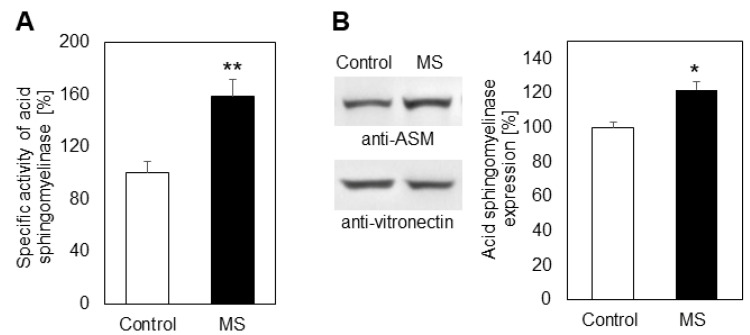
Specific activity (**A**) and protein expression (**B**) of acid sphingomyelinase in plasma from multiple sclerosis (MS) patients (filled bars) and control individuals (empty bars). Values are expressed as percent change of control (100%). Representative images from Western blot analysis with specific antibodies to acid sphingomyelinase (anti-ASM) are shown in the left part of panel (**B**). Reaction with anti-vitronectin antibodies (anti-vitronectin) was used as an internal control for loading. Graphs show pooled data from at least three independent experiments. Values represent means ± SD. Panel (**A**): ** *p* < 0.01; Panel (**B**): * *p* < 0.05.

Since ceramide in erythrocyte membranes and plasma is a product of the secreted acid sphingomyelinase (ASM), we analyzed its activity in blood plasma (Figure 3). The results showed that the enzyme was more active in plasma from MS patients (Figure 3A, increase of about 58%), implying that SM is used more actively as a source of ceramide under these pathological conditions. Additional studies were performed using Western blot analysis to elucidate whether the elevated degradation of SM to ceramide was due only to activation of ASM, or the expression of this protein was also affected in the course of MS progression (Figure 3B). Immunoblotting with specific antibodies showed that the expression of ASM was increased in plasma of MS patients by 22%. Although modest, this rise in the detected protein level was statistically significant (*p* < 0.05). 

**Figure 4:**
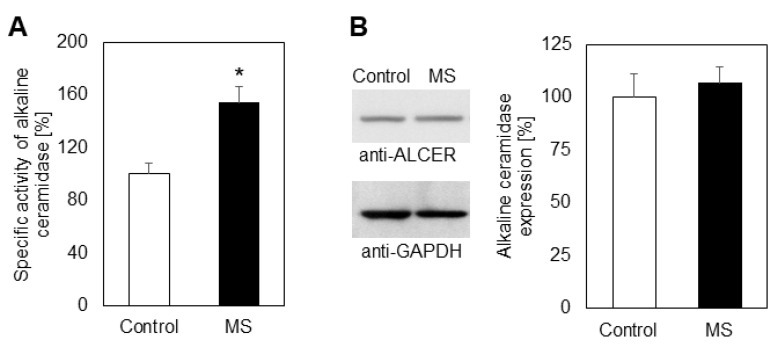
Specific activity (**A**) and protein expression (**B**) of alkaline ceramidase in erythrocyte ghosts from multiple sclerosis (MS) patients (filled bars) and control individuals (empty bars). Values are expressed as percent change of control (100%). Representative images from Western blot analysis obtained with antibodies against alkaline ceramidase (anti-ALCER) are shown on the left part of panel (**B**). Reaction with anti-glyceraldehyde-3-phosphate dehydrogenase antibodies (anti-GAPDH) was used as an internal control for loading. Graphical depiction of the percent change in alkaline ceramidase expression is presented on the right part of panel (**B**). Data represent pooled results from at least three independent experiments. Values represent means ± S.D. * *p* < 0.05. The differences between the values for Alkaline ceramidase expression were not statistically significant.

Thus, it seems likely that both upregulation and higher expression of ASM underlie the elevation of CER, which through ceramidase, yields SPH, the letter being phosphorylated by sphingosine kinase to S1P. To analyze in detail whether the sphingolipid catabolic pathway is responsible for accumulation of sphingosine, we measured the activity of alkaline ceramidase (ALCER), which is located preferentially in erythrocytes [14]. ALCER was activated by 52% in MS ghosts but unlike ASM its expression did not show any detectable difference when compared to control erythrocytes (Figure 4A,B).

**Figure 5:**
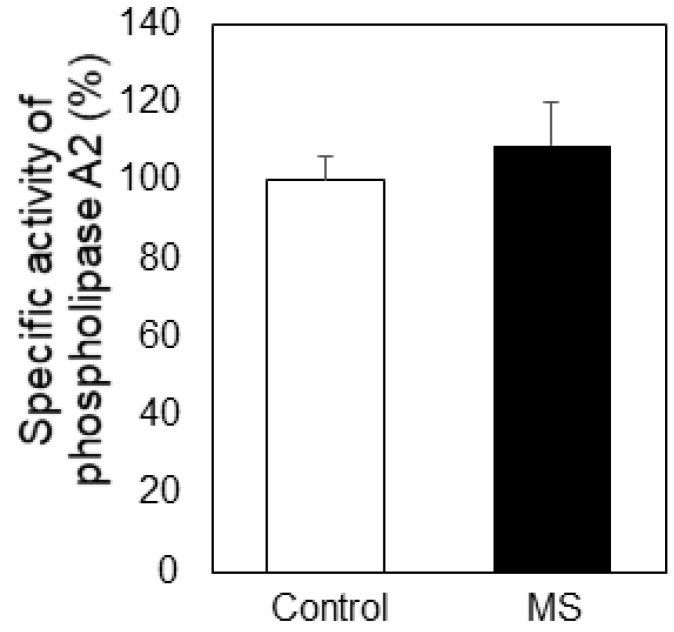
Specific activity of phospholipase A2 (PLA2) in plasma from multiple sclerosis (MS) patients (filled bars) and control individuals (empty bars). Alteration of PLA2 activity is presented as % of control. All measurements were performed in triplicate. Values are means ± SD. The calculated differences are not statistically significant.

The relatively high content of LPC in blood plasma and erythrocytes ghosts of MS patients (Figure 1) focused our attention on the activity of PLA2, because this enzyme produces LPC and unsaturated fatty acids, most commonly AA, which are in constant exchange between plasma and erythrocytes (Figure 5). A tendency of slight activation of PLA2 was observed in MS plasma but the observed differences were not statistically significant (Figure 5).

### 2.4. Fatty Acid Determination in Erythrocyte Membranes and Plasma from Multiple Sclerosis Patients

Our studies confirmed the already known fact, that the level of saturated fatty acids (SAT) was higher in erythrocytes and plasma of MS patients (Table 3). We compared the ratios between SAT, UNSAT and polyunsaturated fatty acids (PUFA) and tried to establish correlations between the obtained values, which could serve as markers, distinguishing MS erythrocytes and plasma from controls. The calculations showed that the UNSAT/SAT ratio was higher than 1.5 in control erythrocytes and lower than 1.5 in MS, implying that the value of 1.5 could serve as a reference value. Similarly, this ratio was higher than 2.0 in control plasma and lower than 2.0 in MS plasma (Table 3). The differences between other ratios such as PUFA/SAT and MONO/SAT were less pronounced, which makes them less likely to serve as markers to distinguish between controls and MS. Thus, we suggest that the UNSAT/SAT ratio could be a useful parameter to distinguish blood from control and MS patients.

### 2.5. Measurement of Lipid Peroxide and Isoprostane Levels in Plasma from Multiple Sclerosis Patients

SM acts as an intrinsic antioxidant which prevents the PL fatty acids from oxidative destruction due to its tight association with the acyl chains. Thus, its reduction could render the polyunsaturated acyl chains more vulnerable to oxidative attack. As already mentioned, oxidative stress is considered as a pathogenic factor, participating in the onset and progression of MS [18]. Studies were carried out on the oxidative destruction of the lipid molecules in plasma from MS and control individuals using lipid-derived markers. The changes in the plasma lipid peroxides (LPO) level of the MS patients are shown in Table 4. The results indicated a significant increase of about 148% of the LPO values in MS plasma compared to control values. Another specific marker that indicates the degree of lipid oxidation is the level of F2-isoprostanes, also referred to as 8-iso-PGF_2α_. These are prostaglandin F_2α_-like compounds, which are produced by free radical–catalyzed peroxidation of arachidonic acid and are assumed as markers of in vivo oxidative stress. As evident from Table 4, the level of isoprostanes was significantly higher (181%) in the plasma of the MS patients. The alterations in isoprostanes level were more pronounced compared to LPO (181% vs. 148%).

## 3. Discussion

Multiple sclerosis (MS) is an autoimmune, inflammatory, degenerative disease of the central nervous system, where the body’s immune system attacks the nerves and the myelin sheath. These processes result in a variety of symptoms involving disturbed movement, fatigue, pain, changes in vision, cognitive difficulties, etc. [26]. The etiology of MS is still unclear but genetic, viral and lifestyle factors are largely suspected [27]. Although there is no ultimate cure for MS, some therapeutic approaches and nutritional schemes are proposed [28] and in some cases dietary alterations focused on lipid supplementation have shown promising results [29,30]. 

Sphingolipids, together with glycerophospholipids, are involved in many cellular functions, including cell proliferation, signaling cascades, apoptosis, etc. Changes in lipid metabolism and level of separate lipid molecular species have been suggested to play important roles in multiple sclerosis pathophysiology and progression. Especially sphingolipids, which are largely expressed in the Central Nervous System (CNS), have been implicated in the pathogenesis of MS [15]. The major sphingolipids are represented by SM, CER, SPH and S1P [31]. They build up specific cascades, in which one component emerges from the other by sequential enzymatic degradation [32]. 

In this work we analyzed the lipid composition of erythrocyte membranes (ghosts) and plasma from MS patients and healthy controls (Figure 1A,B). Although there are reports showing such results, some of them are controversial [33,34,35], which is why we analyzed the lipid alterations induced by MS. Some have reported a decrease and others an increase of major lipids of significant physiological importance, such as SM and PC. It is possible that the observed differences between our results and the mentioned reports are due to methodological differences or to the use of whole erythrocytes, instead of erythrocyte membranes. As evident from Figure 1, the choline-containing phospholipid SM was reduced, these changes being more pronounced in erythrocyte membranes. We were interested mainly in the alterations in this first member of the sphingolipid cascade, because it is a major component of erythrocyte membranes, participating in raft formation and also acting as a main source of CER, SPH and S1P. Its decrease occurs through degradation by acid sphingomyelinase (ASMase), which produces phosphorylcholine and a sphingolipid of significant physiological importance, CER. There are reports demonstrating that CER regulates the function of phospholipase A2 [36] and some phosphatases [37]. Our results showed that the level of CER was elevated in both the plasma and erythrocyte ghosts (Figure 2A,B), which is in accordance with the upregulated ASM circulating in MS plasma (Figure 3A). Additional studies were performed using Western blot analysis to elucidate whether the elevated degradation of SM to ceramide was due only to activation of ASM, or the expression of this protein was also affected in the course of MS progression. Western blot showed that the expression of ASM was elevated by 22% in MS plasma (Figure 3B), indicating that ASM expression was also a factor, underlying the stimulated degradation of SM.

Further research was focused on the accumulation of SPH, which is precursor of S1P, the latter being the most functionally active member of sphingolipid metabolites. A large number of studies has been concentrated on S1P and much less interest has been devoted to SPH as a member of the sphingolipid family. This is why we studied the MS-induced changes in the mechanism of SPH production, which occurs through degradation of CER by alkaline ceramidase (ALCER), the latter being reported in human erythrocytes [14]. Our studies showed that the content of SPH was elevated in both erythrocytes and plasma of MS patients (Figure 2C,D). ALCER was up-regulated in MS ghosts compared to controls and Western blot analysis showed that MS progression did not affect the expression of erythrocyte ALCER.

The major membrane phospholipid, PC, which serves as PLA2 substrate, showed a trend for reduction in both erythrocyte membranes and plasma from MS patients, although the observed differences were not statistically significant (Figure 1A,B). In addition, LPC, was elevated in MS erythrocytes and plasma. LPC, together with AA, are products of PLA2. There is evidence that the metabolic pathway of AA is activated in the central nervous system of MS patients [38]. AA is released from the membrane PLs by PLA2, the latter being modulated by elevated concentrations of ROS [39]. In turn, released AA via cyclooxygenases and lipoxygenases produces pro-inflammatory thromboxanes and leukotrienes, which are accumulated in MS patients [38]. The mentioned above derivatives of AA are proposed to be involved in the pathogenesis of demyelination and axonal disturbance, thus contributing to progression and aggravation of motor disabilities. There are reports demonstrating that CSF and post-mortem brains of MS patients show augmented levels of the AA metabolic pathway [38]. What is more, there is evidence that prostaglandins and leukotrienes are increased in the CSF of MS patients [40,41].

As mentioned above PUFA are products of PLA2, the latter circulating in human plasma. PLA2 plays critical roles in the pathogenesis of neurodegenerative diseases such as multiple sclerosis by enhancing oxidative stress and initiating inflammation. The levels of PLA2 activity in MS patients and the effect of inhibiting PLA2 on the severity in different experimental models of neurodegenerative pathologies have not been elucidated. Inhibiting sPLA2 leads to lower clinical severity or no signs of experimental autoimmune encephalomyelitis (EAE) in mice, and a lower incidence of EAE lesions compared to animals without PLA2 inhibition [42]. The same authors reported that measurement of PLA2 activity in patients with MS and controls showed no significant difference between groups, except when PLA2 activity was measured in urine. Our studies showed a slight tendency for elevation of PLA2 activity in plasma of MS patients but the differences were statistically insignificant (Figure 5). This observation correlated with the slight decrease of PC (Figure 1A,B), which acts as PLA2 substrate. The level of the other PLA2 product, AA, was lower in MS plasma, which could be possibly explained by a quick engagement of this PUFA in the synthesis of pro-inflammatory products like prostaglandins and leukotrienes in MS patients. AA reduction could also be due to its extensive oxidative degradation in MS plasma. It contains four double bonds in its molecule which makes it an excellent target for oxidative attack.

Changes in plasma FA have been reported to correlate with the progression of MS. Most epidemiological studies state that diets rich in saturated FA correlate with MS progression, provoking the development of this pathology. On the other hand, diets rich in PUFA seem to decrease the risk of MS development [6], may even ameliorate MS symptoms and are related to the mechanisms of disease development [43,44,45]. Our studies confirmed the above-mentioned observation, that the level of saturated fatty acids (SAT) is higher in erythrocytes and plasma of MS patients. We compared the ratios between saturated (SAT), unsaturated (UNSAT) and PUFA and tried to establish correlations between the obtained values, which could serve as markers, distinguishing erythrocytes and plasma of MS from controls. The results showed that the UNSAT/SAT ratio was higher than 1.5 in control erythrocytes and lower than 1.5 in MS. Similarly, this ratio was higher than 2.0 in control plasma and lower than 2.0 in MS plasma (Table 3). The other analyzed ratios such as PUFA/SAT and MONO/SAT did not show so pronounced differences which could make them suitable markers to differentiate between blood of healthy individuals and MS patients.

Reports show that not only unsaturated fatty acid products, but also CERs play role in development of oxidative stress. There is evidence that CERs can significantly increase reactive oxygen species liberation in glial cells [46]. MS pathogenesis is closely related to oxidative stress and free oxygen radicals which, together with pro-inflammatory mediators, underlie the disease onset and progression [47,48]. High content of ROS has been suggested to destroy the blood–brain barrier and consequently increase the migration of monocytes to CNS, thus inducing focal inflammation and demyelination [18,49]. Augmented levels of advanced oxidation protein products and oxidized glutathione have been reported in blood plasma of MS patients [50]. The CNS contains high levels of PUFA and shows high oxygen consumption, which is a prerequisite for excessive formation of lipid peroxides that impair the brain tissue in patients with MS [51,52]. High levels of lipid peroxidation markers were reported in blood plasma of MS patients [23,53].

We measured the plasma content of lipid-derived markers indicating oxidative destruction such as lipid peroxides and F2-isoprostanes (Table 4). The content of 8-iso-prostaglandin F2α (8-iso-PGF2α) is recognized as a reliable biomarker of lipid peroxidation and oxidative stress [53]. As evident from Table 4, both markers of lipid oxidative modification were higher in MS patients compared to controls. In accordance with our results, other authors also observed elevated content of lipid peroxidation markers in plasma of patients with MS [23,51]. Also, Mir et al. [54] reported higher levels of isoprostanes in CSF of MS patients. Isoprostanes represent a class of lipid peroxidation products that are generated upon oxidative attack on AA, which is an acyl chain component of the membrane phospholipids [55,56,57]. The significance of oxidative stress in MS progression implies that the search of adequate therapeutic approaches which induce reduction of the overall oxidative stress is of particular importance. 

There are certain limitations concerning the conclusions drawn, which are related mainly to the restricted number of the tested cohort. The reported results were observed in patients with relatively high disability and long disease duration. Further studies should be performed to confirm the validity of the present results for patients with lower disability and disease duration.

In conclusion, multiple factors are reported to increase the risk of onset and progression of multiple sclerosis, but the etiology of this pathology remains largely unclear. As typical for other inflammatory neurodegenerative pathologies, oxidative stress and lipid peroxidation are closely related to MS development. Alterations in the lipid profile seem to be specific for this disease which is associated with dysregulation of the lipid homeostasis and lipid metabolism, this being valid especially for sphingolipids. Lipid analysis presented in this work demonstrates the changes of lipid molecules and their metabolism in erythrocytes and plasma of MS patients. Clinical lipidomics has the potential to be applied in MS diagnosis as well as in evaluation of therapeutic approaches by providing a detailed analysis of the lipidome profile of MS patients. Finally, clinical lipidomic analytical approaches could become a promising contemporary tool for personalized medicine.

## 4. Materials and Methods

### 4.1. MS Patients and Control Individuals

All patients and healthy individuals gave written informed consent before being included in this study. The designed investigations were approved by the local ethics commission. Eighteen healthy non-smoking individuals (nine men and nine women) in the range 35–57 years of age participated in the investigations. The 18 patients (nine men and nine women), aged 34–59 years were clinically diagnosed with relapsing-remitting multiple sclerosis (the most common form of MS), according to the McDonald revised diagnostic criteria for MS (2017) and by neuroimaging. The degree of disability was determined according to the scale of Kurtzke’s Extended Disability Status Scale (EDSS) [58]. The MS patients were free of disease-modifying therapy three months prior to blood sample collection.

### 4.2. Blood Samples Collection and Preparation of Erythrocyte Ghosts

Blood samples of 10 mL were collected by venipuncture of the peripheral forearm vein around 8 AM after overnight fasting. The obtained blood was anticoagulated with sodium citrate. The erythrocytes were pelleted by centrifugation at 2000× *g* for 10 min at 4 °C. The supernatant from this spin was centrifuged at 10,000× *g* for 10 min at 4 °C to pellet any remaining cells or platelets. The supernatant thus obtained was used in the experiments listed below. Ghosts were prepared by freezing and thawing the red cells. Any intact cells which remained after three times freezing and thawing were pelleted by centrifugation. Erythrocyte membranes were kept frozen at −70 °C until used.

### 4.3. Lipid Extraction and Analysis

Lipids from erythrocyte ghosts and plasma were extracted with chloroform/methanol according to the procedure of Bligh and Dyer [59]. The organic phase was concentrated and analyzed by HPLC (WATERS Alliance 2695).

### 4.4. Fatty Acid Analysis 

The phospholipid extracts were saponified with 0.5 N methanolic KOH and methylated with boron trifluoride-methanol complex (Merck, Darmstadt, Germany). The fatty acid methyl esters were separated by GC-MS on a column 60 m × 0.25 mm ID BPX70 × 0.25 µm.

### 4.5. Determination of Ceramide 

Separation of NBD-ceramide was performed on a disposable reverse phase column (Nova-Pack, C18) using methanol: water: 85% phosphoric acid (850:150:0.125 *v*/*v*) at flow rate of 2 mL/min. The HPLC was equipped with an automatic injector with an injection loop between 50 and 1000 μL. Under these conditions, the typical elution time for NBD-ceramide was about 10 min. The excitation wavelength was 455 nm and emission was detected at 533 nm [60].

An alternative method was also applied for determination of ceramide level using Ceramide ELISA kit (MyBioSource, Catalog No: 3804520) according to the manufacturer’s instructions.

### 4.6. Determination of Sphingosine 

Plasma sphingosine level was determined using sphingosine ELISA kit (Aviva Systems Biology, San Diego, CA, USA, Catalog No: OKEH02615) according to the manufacturer’s instructions.

### 4.7. Acid Sphingomyelinase (ASM) Activity Assay

ASM activity in plasma was measured using NBD-SM as substrate. Before use, the substrate was dried under nitrogen, resuspended in 200 mM sodium acetate (pH 5.0) and sonicated for 10 min to obtain micelles. Incubations were performed for 30 min at a final volume of 0.5 mL. The reaction was terminated by extraction in 0.5 mL CHCl_3_/CH_3_OH (2:1, *v*/*v*). The samples were vortexed for 10 s and centrifuged at 5000× *g*. An aliquot of the aqueous phase was measured for fluorescence. The hydrolysis of NBD-SM by ASM results in release of NBD-phosphocholine into the aqueous phase, whereas ceramide and the unreacted NBD-SM remain in the organic phase. Results were expressed as pmol hydrolyzed SM/min/µL plasma [60].

### 4.8. Alkaline Ceramidase (ALCER) Activity Assay 

Ceramidase activity was determined by the release of sphingosine from ceramide. Briefly, erythrocyte ghosts (50 µg protein) were incubated with ceramide substrate (100 mM) in buffer 25 mM Tris-HCl bufer, pH 9, containing 5 mM CaCl_2_ and 5 mM MgCl_2_) at 37 °C for 30 min. The reaction was terminated by addition of 0.5 mL CHCl_3_/CH_3_OH (2:1, *v*/*v*). After the extraction, sphingosine was determined by sphingosine ELISA kit (Aviva Systems Biology, Catalog No: OKEH02615). Results were expressed as pmol hydrolyzed CER/min/µg ghost protein [60].

### 4.9. Phospholipase A2 Assay

Phospholipase A2 activity was assayed by the method described below. Plasma samples were incubated with 100 nmol egg yolk phosphatidylcholine as substrate in 100 mM Tris-HCl pH 8.6 with 5mM CaCl_2_ and 0.1% fatty acid free bovine serum albumin. Incubation was carried out for 30 min at 37 °C with continuous shaking in a total volume of 0.5 mL. The reaction was stopped with 0.5 mL chloroform/methanol (2:1 *v*/*v*) and the liberated fatty acids were extracted. After methylation the fatty acid methyl esters were determined by GC-MS. PLA2 activity was calculated as pmol fatty acids/min/µL plasma and the alteration was expressed as % compared to control values.

### 4.10. Determination of Lipid Peroxides

Lipid peroxidation in plasma was measured by a fluorimetric method using thiobarbituric acid (TBA). The plasma lipids containing lipid peroxides were precipitated with phosphotungustic acid and then were incubated with TBA. The fluorescence of the reaction product was measured with excitation at 515 nm and emission at 553 nm. The concentration of lipid peroxides was expressed in terms of malondialdehyde (ng/mL plasma) using tetramethoxypropane as a standard.

### 4.11. Determination of 8-ISO-PGF_2α_

The 8-iso-PGF_2α_ levels were determined using ELISA isoprostane kit ABCAM, (ab175819) according to the manufacturer’s directions.

### 4.12. Western Blotting and Antibodies

Prior to electrophoresis, plasma samples were depleted from albumin and IgG by using Albumin IgG Depletion Spintrap^™^ (Merck, GE28-9480-20) according to the manufacturer’s instruction. Erythrocyte ghosts and plasma samples (15–20 µg was mixed with equal volume of 5× sample buffer (60 mM Tris-HCl, pH 6.8, 2% SDS, 10% glycerol, 5% β-mercaptoethanol and bromophenol blue) and heated for 4 min at 95°C. Proteins were resolved on SDS-PAGE, transferred to nitrocellulose membrane and blocked for 1 h in 5% non-fat dry milk in TBST (50 mM Tris base, 200 mM NaCl, 0.1% Tween-20, pH 7.4). Membranes were than incubated with the appropriate primary and secondary antibodies, including polyclonal anti-alkaline ceramidase 2 (A09706-1, Boster Bio, Pleasanton, CA, USA), monoclonal anti-acid sphingomyelinase (ab272729, Abcam, Cambridge, UK), monoclonal anti-vitronectin (D8, Santa Cruz, Dallas, TX, USA), monoclonal anti-GAPDH (sc-32233, Santa Cruz), anti-mouse IgG (Fab) HRP conjugate (SAB-100, Stressgen), anti-rabbit IgG (Fab) HRP conjugate (SAB-300, Stressgen). Immunoblots were visualized using the ECL system (Santa Cruz).

### 4.13. Protein Determination

The content of protein was determined according to the method of Bradford and colleagues [61].

### 4.14. Statistical Analysis

Statistical processing of the data was made by one-way analysis of variance (ANOVA), using In Stat software, Graph Pad In Stat 3.1, developed by Graph Pad Software, San Diego, CA, USA.

## 5. Conclusions

1. The first member of the sphingolipid cascade, sphingomyelin (SM), is reduced and ceramide (CER) is increased in erythrocyte membranes and plasma of MS patients.
2. The expression analyzed by Western blot and the activity of acid sphingomyelinase (ASM) were elevated in plasma of MS patients compared to controls.
3. The activity of alkaline ceramidase (ALCER) is upregulated but the expression is unchanged in erythrocyte membranes of MS patients compared to controls.
4. We suggest that the unsaturated/saturated (UNSAT/SAT) fatty acid ratio could serve as a marker to differentiate between erythrocytes and plasma of MS patients and controls.
5. Our results confirmed that the lipid-derived markers of oxidative stress, lipid peroxides and isoprostanes, are higher in the plasma of MS patients than in control individuals.

## Figures and Tables

**Table 1:** Values of Kurtzke’s Expanded Disability Status Scale (EDSS).

Male		Female	
Patient 1	5	Patient 10	4
Patient 2	4	Patient 11	6
Patient 3	6	Patient 12	6
Patient 4	5	Patient 13	5
Patient 5	5	Patient 14	5
Patient 6	7	Patient 15	7
Patient 7	6	Patient 16	7
Patient 8	6	Patient 17	6
Patient 9	7	Patient 18	7

**Table 2:** Characteristics of patients with multiple sclerosis and control individuals.

Male/female ratio (patients)	9:9
Male/female ratio (controls)	9:9
Age range (patients)	34–59
Age range (controls)	35–57
Weight (kg) (patients)	71 ± 11
Weight (kg) (controls)	74 ± 12
Duration (years) (patients)	16 ± 5
Duration (years) (controls)	n.a.
BMI (patients)	23.3 ± 2.1
BMI (controls)	25.8 ± 2.6

**Table 3:** Fatty acid composition of erythrocyte ghosts and plasma from control individuals and multiple sclerosis (MS) patients (% of total fatty acids).

Fatty Acids	Erythrocytes		Plasma	
	Control	MS	Control	MS
C 16:0	24.1	29.9 **	20.5	23.3 *
C 18:0	12.8	17.1 **	9.9	12.7 *
C 18:1	20.2	17.7 *	16.7	16.7
C 18:2	15.9	12.5 **	25.4	22.8 *
C 20:3	1.5	1.3	n.d.	n.d.
C 20:4	18.4	17	17.5	17.9
C 22:4	1.2	0.7 *	2.6	1.5 *
C 22:6	5.9	3.8 *	7.4	5.1 *
UNSAT/SAT	1.71	1.127	2.292	1.782
PUFA/SAT	1.162	0.751	1.74	1.317
MONO/SAT	0.5	0.413	0.552	0.465

UNSAT-unsaturated fatty acids; SAT-saturated fatty acids; PUFA-polyunsaturated fatty acids; MONO-monounsaturated fatty acids. ** *p* < 0.01; * *p* < 0.05.

**Table 4:** Alterations in the level of lipid-derived markers of oxidative stress LPO and 8-isoprostanes in plasma from MS patients. LPO-lipid peroxides, presented as ng/mL; 8-isoprostanes are presented as pg/mL, * *p* < 0.001.

Markers	Control	MS	% Alteration
LPO	9.7	14.4 *	148
F2-isoprostanes	41.2	74.5 *	181

## Data Availability

The datasets generated during the current study are available from the corresponding author on reasonable request.
